# Using Bioassays and Species Sensitivity Distributions to Assess Herbicide Toxicity towards Benthic Diatoms

**DOI:** 10.1371/journal.pone.0044458

**Published:** 2012-08-30

**Authors:** Floriane Larras, Agnès Bouchez, Frédéric Rimet, Bernard Montuelle

**Affiliations:** Institut National de la Recherche Agronomique, UMR 0042, Carrtel, Thonon, France; Federal University of Rio de Janeiro, Brazil

## Abstract

Although benthic diatoms are widely used in ecological studies of aquatic systems, there is still a dearth of data concerning species sensitivities towards several contaminants. Within the same community, different species may respond differently depending on their physiological and ecological characteristics. This lack of knowledge makes specific appropriate risk assessment impossible. To find out whether species sensitivity distribution (SSD) could be used to estimate the risk of herbicide toxicity for diatoms, we need to know whether their sensitivity depends on their physiological and ecological characteristics. We carried out single-species bioassays on 11 diatom species exposed to 8 herbicides. Dose-responses relationships were used to extrapolate the Effective Concentration 5 (EC_5_) and the Effective Concentration 50 (EC_50_) for each exposure. These data were used to fit a SSD curve for each herbicide, and to determine the Hazardous concentration 5 (HC_5_) and 50 (HC_50_). Our results revealed a high level of variability of the sensitivity in the set of species tested. For photosystem-II inhibitor (PSII) herbicides, diatoms species displayed a typical grouping of sensitivity levels consistent with their trophic mode and their ecological guild. N-heterotroph and “motile” guild species were more tolerant of PSII inhibitors, while N-autotroph and “low profile” guild species were more sensitive. Comprehensive SSD curves were obtained for 5 herbicides, but not for sulfonylurea herbicides or for dimetachlor, which had toxicity levels that were below the range of concentration tested. The SSD curves provided the following ranking of toxicity: diuron> terbutryn> isoproturon> atrazine> metolachlor. The HC that affected 5% of the species revealed that, even at the usual environmental concentrations of herbicides, diatom assemblages could be affected, especially by isoproturon, terbutryn, and diuron.

## Introduction

Rivers and lakes are often compromised by contaminants such as pesticides derived from watershed runoff or urban discharges [1]–[3]. In particular, the coastal zones of lakes are exposed to higher concentrations of toxicants than the pelagic zone [4], leading to a higher exposure of organisms in these zones. Among the various herbicides found in water bodies, atrazine (s-triazines), diuron and isoproturon (phenylureas) are some of the main substances monitored in France [5]. Some of these herbicides are banned or restricted, but they are still found in European inland water ecosystems, and sometimes reach concentrations above the Annual Average- Environmental Quality Standard (AA-EQS) defined in the Water Framework Directive [2], [5]–[7]. In small rivers, ponds, and coastal zones of lakes, microalgae and especially diatoms are crucial primary producers and account for a considerable proportion of the fixed biomass as well as carrying out some of the main biochemical processes. Benthic diatoms are known to be good indicators of ecosystem water quality, especially of the trophic level and physical disturbances [8], [9].

The impact of herbicides, such as triazines and phenylureas, on phototrophic organisms is well known. They inhibit photosynthesis by disrupting the electron transmission chain in the thylakoid membrane, leading to the dissipation of luminous energy [10]. As a consequence, benthic diatoms are potential targets due to the phytotoxic characteristics of these herbicides [10], [11]. Other herbicides belonging chloroacetamide and sulfonylurea families are also found in water. For example, metolachlor was found in 37% of stations analyzed station in the Adour-Garonne basin in France [7]. They act on cells by inhibiting the biosynthesis of very long chain fatty acids or the biosynthesis of amino-acids [11], [12]. Some authors have already demonstrated their phytotoxicity on microalgae [13]–[15].

Single-species laboratory bioassays for microalgae and herbicides are mostly carried on phytoplanktonic species, such as *Scenedesmus* spp. or *Chlorella* spp., which are easy to grow under controlled laboratory conditions. A few studies have investigated the sensitivity of benthic diatoms under single-species conditions. These studies showed that a) the sensitivity towards the same herbicide is very variables in different species [16]–[18] and b) pre-exposure of diatoms can increase their herbicide tolerance [18]. Laboratory single-species bioassays provide usefull data for assessing individual sensitivities, however in the natural environment, herbicides could disrupt communities in which many species are present and which may interact and respond differently, depending on their physiological and chemical parameters [19]. The variation of species sensitivity in a community or an assemblage towards one or several toxicants can be expressed in terms of a cumulative distribution known as the Species Sensitivity Distribution (SSD) [20]. Such distributions are based on selected benchmarks obtained from single-species bioassays. SSDs are used in risk assessment, as predictive models, and to derive thresholds in order to protect environmental biodiversity. The threshold concentration is often defined as the Hazardous Concentration for 5% of the species (HC_5_), which is expected to spare 95% of the species in the assemblage [20], [21].

Benthic diatoms are known to dominate biofilms of benthic littoral zones in terms of abundance [22], [23]. These algae present a great diversity, which enabled some authors to develop various biotic indices for nutrient and organic matter assessment in rivers and lakes [24], [25]. Within this great diversity, species exhibit various sensitivity to herbicides [14], [16], [26]. Even if others sorts of algae, as chlorophytes and cyanobacteria are more sensitive [27], they are not as abundant as diatoms which appeared as relevant and representative organisms for the benthic zone. With the view to assess herbicides risk assessment of benthic littoral zone, benthic diatoms appeared then as ecologically relevant tools.

The two aims of this study were a) to determine the variation of the sensitivity of benthic diatom species towards herbicides in an artificial assemblage and b) to find out whether SSD models based on benthic diatoms sensitivities can be used to find out whether herbicides constitute a risk at concentrations found in the environment. In this study we develop a data base of EC_5_ and EC_50_ thresholds based on single-species bioassays for 11 benthic diatoms exposed to 8 agricultural herbicides. These two thresholds were chosen because of their environmental relevance and because they are widely used in the literature, which made it easier to compare our results with those of other studies. These EC_5_ and EC_50_ values were then used to construct SSD models in order to derive HC_5_ values for a diatom assemblage. Experiments were carried out as single-species bioassays, using diatom strains derived from benthic biofilms (taken from the Geneva Lake coastal zone) or from the INRA Thonon Culture Collection. The selected endpoint was the growth rate calculated from the chlorophyll a fluorescence.

## Methods

### Benthic diatoms cultures

Eleven species of diatom were selected from the species found in biofilms from the benthic coastal zone of Lake Geneva and according to the literature (Table 1). Strain collection in Lake Geneva was made on public area, not requiring any specific permission, and did not involve protected species. In order to ensure that we were working on a diatom diversity representative of freshwater benthic ecosystems, we selected diatoms with a variety of life forms and taxonomic diversity. In natural biofilms, pennate diatoms outnumber centric diatoms. Consequently, most of the species tested were pennate and only one was centric (*Cyclotella meneghiniana*, CMEN). Most of them are classified as benthic, and in fact all had been isolated from benthic biofilms and came from the Thonon Culture collection (Thonon-Les-Bains, France, http://www.inra.fr/carrtel-collection). The diatoms selected for this study were: *Fragilaria capucina* var. *vaucheriae* (FCVA), *Fragilaria rumpens* (FRUM), *Fragilaria ulna* (FULN), *Craticula accomoda* (CRAC), *Mayamaea fossalis* (MAFO), *Eolimna minima* (EOMI), *Nitzschia palea* (NPAL), *Achnanthidium minutissimum* (ADMI), *Cyclotella meneghiniana* (CMEN), *Encyonema silesiacum* (ESLE) and *Gomphonema parvulum* (GPAR). Cultures were maintained in DV culture media (http://www6.inra.fr/carrtel-collection_eng/Culture-media/Composition-of-the-culture-media) filtered at 0.22 µm (Millipore). They were grown in a 300 mL Erlenmeyer flask at 21±2°C, and with a 16:8h light:dark cycle at 66 µmol. m^−2^. sec^−1^.

**Table 1:** Characteristics of the 11 diatom species: taxonomy, references, and ecological guild.

Order	Species	Code^a^	TCC^b^	Ecological guild^c^
Naviculales	*Craticula accomoda*, (Hustedt) D.G. Mann	CRAC	107	Motile
	*Eolimna minima*, (Grunow) Lange-Bertalot	EOMI	524	Motile
	*Mayamaea fossalis*, (Krasske) Lange-Bertalot	MAFO	366	Motile
Cymbellales	*Encyonema silesiacum*, (Bleisch) D.G. Mann	ESLE	678	Low profile
	*Gomphonema parvulum*, (Kützing) Kützing	GPAR	653	High profile
Fragilariales	*Fragilaria capucina* var. *vaucheriae*, (Kützing) Lange-Bertalot	FCVA	752	High profile
	*Fragilaria ulna*, (Nitzsch) Lange-Bertalot	FULN	365	High profile
	*Fragilaria rumpens* (Kützing) G.W.F. Carlson	FRUM	666	High profile
Bacillariales	*Nitzschia palea*, (Kützing) W. Smith	NPAL	139–2	Motile
Achnantes	*Achnanthidium minutissimum*, (Kützing) Czarnecki	ADMI	746	Low profile
Thalassiosirales	*Cyclotella meneghiniana*, Kützing	CMEN	755	Low profile

a Species code in the OMNIDIA database [48]. ^b^Strain reference in the INRA Thonon Culture Collection. ^c^Ecological guild from Passy [42] and refined by Berthon [46].

### Herbicides

The herbicides used in this study have all been detected in the coastal zone of Lake Geneva, and were selected on the basis of toxicity and mode of action (Table 2). They had all been detected regularly in the Lake since 2004 [4].We used atrazine (purity 99.9%), terbutryn (purity 99.3%), diuron (purity 99.5%), isoproturon (purity 99.9%), metolachlor (purity 98%), dimetachlor (purity 99.9%), amidosulfuron (purity 99.9%) and foramsulfuron (purity 97.5%). These substances were obtained from Sigma-Aldrich (St Louis, MO 63103, USA). They are representative of 4 different modes of action and have different Kow values (Table 2).

**Table 2:** Chemical properties and mode of action of the 8 herbicides.

Family	Herbicide	Mode of action	Log Kow	Solubility (mg/l)
Phenylureas	Diuron	Photosystem 2 inhibition (site B)	2.87	35.6
	Isoproturon		2.5	70.2
Triazines	Atrazine	Photosystem 2 inhibition (site A)	2.7	35
	Terbutryn		3.65	25
Chloroacetamides	Metolachlor	Inhibition of the synthesis of very long chain fatty acid	3.4	530
	Dimetachlor		2.17	2300
Sulfonylureas	Foramsulfuron	Growth regulators (higher plants)	−1.56	3293
	Amidosulfuron		−0.78	3070

### Bioassays

Herbicide stock solutions were prepared by dissolving herbicides in DV growth media before filtering on a 0.22 µm filter unit (Millipore) and stored at 4°C in the dark. Due to their low solubility, atrazine and diuron were first dissolved in 0.05% Dimethyl sulfoxide (DMSO), and then sonicated for 30 minutes. The non-toxicity of 0.05% DMSO dissolved in DV media was confirmed on diatoms before the bioassays (data not shown).

The 11 diatom species were exposed to the 8 herbicides in single-species laboratory bioassays (11diatoms*8 herbicides, i.e. 88 bioassays), and their growth rates were measured as an endpoint. Bioassays were carried out in triplicate in 30 mL glass tubes, with a cap that permits gaseous exchange. Each tube contained 1 mL of a contaminated solution and 1 mL of an 8 to 10 day old strain culture in the exponential growth phase. The range of concentrations to be used in bioassays for each species and each herbicide had previously been determined by means of preliminary tests in 96 well microplates. At the start of the experiment, cell density was approximately 10000±2000 cells/mL according to the Standard Guide for Conducting Static Toxicity Tests with Microalgae [28]. Seven to 10 pesticide concentrations were tested, and each of the diatoms were exposed for 96 h under the same experimental conditions as used for the cultures. At the end of the experiment, each tube was shaken (MS2 Minishaker, IKA) for a few seconds to resuspend the algae in the media, and to homogenize the solution. The remaining fixed algae were carefully scraped off with a rod. From each tube, 250 µL of solution was transferred into a 96 well black microplate in quadruplicate. The growth rate was determined by measuring the chlorophyll a fluorescence and was quantified in Reference Fluorescence Units (RFUs) using a Fluoroskan (Fluoroskan Ascent,Thermo-Scientific,Finland) with a 430 nm excitation filter and a 680 nm emission filter after incubating for 20 minutes in the dark. To assess the reference growth of diatoms during assay, the fluorescence was checked at the beginning and at the end of the experiment in the control. The linear correlation between chlorophyll a fluorescence (measured using the Fluoroskan) and the cell (optically counted) had previously been checked on the basis of 5 measurements for each species. As chl a fluorescence is correlated with cell density, the growth rate was then calculated as described by Debenest et al.[29] for cell density: 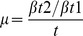



Where *βt2* and *βt1* represent the fluorescence at the end and at the beginning of the experiment respectively. *t* is the exposure time, in days.

### Data analysis

#### Dose-response modeling

From each single-species bioassay, the dose-response curve was fitted with R software and the “drc” package [30]. Two non-linear regression models were used depending on the features of the dose-response curve. In the case of a monotically-decreasing function, a log-logistic model (equation 1) was applied where *d* was the upper limit of the curve, *c* was the lower limit, and *b* was the relative slope around the EC_50_ known as *e* [30].

Equation 1: 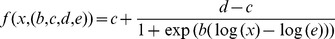


Hormesis, or the “greening-effect”, leads to a biphasic dose-response in which curves have a typical inverted U-shape or J-shape [31]. In the context of hormesis, the Cedergreen-Ritz-Streibig model (equation 2) was used to fit the data better. This model considered the stimulation phase as the *f* parameter [32]. The higher *f*, the greater the hormesis effect.

Equation 2: 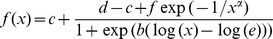



The Effective Concentration (EC) affecting the growth of 5% (EC_5_) or 50% (EC_50_) of the population was numerically determined from each dose-response curve. For low effect levels, many ECs are used (e.g.:NOEC, LOEC, EC_10_, EC_20_). In this study, we preferred to work with the EC_5_, because in most of the tests this threshold fell between two concentrations tested. This meant that EC_5_ was a reliable value which could be determined from the regression curve. For each herbicide, 11 EC_5_ and 11 EC_50_ values were expected. Their 95% confidence intervals were calculated using bootstrap methods.

#### Species Sensitivity Distribution modeling

SSD curves were fitted with R software using the EC values derived from dose-response curves. For each herbicide, two kinds of SSD curves were fitted: the SSD-EC_5_ and the SSD-EC_50_, which were based on the 11 EC_5_ and the 11 EC_50_ values, respectively. From each SSD-ECx curve, the slope and 2 Hazardous Concentrations (HC_5_, HC_50_) were numerically derived. They correspond to the herbicide concentrations affecting 5% and 50% of the species in an assemblage, respectively.

For each herbicide *i*, the SSD-EC_50i_ curve was fitted using log-logistic regression (equation 3).Several distributions are proposed to fit the SSD model, but in this study the criterion used was the best fit of the data. For a small dataset there is no significant difference between log normal and log-logistic regression [33] and so, as some studies have already shown that the log logistic regression fits the data best [34], [35] we chose to use this. According to Chèvre et al [36], *HC50_EC50,i_* was the hazardous concentration affecting 50% of species, and *EC50_i,s_* was the EC_50_ of herbicide *i* for species *s*.

Equation 3: 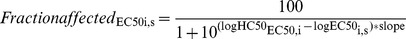



## Results

### Individual dose-response relationship

In this study, 88 bioassays were carried out and the results are presented on Table 3. Thirty-five of these tests failed to provide reliable EC estimations for various reasons. According to this endpoint, the highest concentrations tested did not produce enough effect to allowed us to fit reliable dose-response curves for the sulfonylurea family. For the chloroacetamide family, only 3 and 7 species, respectively, had an EC_50_ below this concentration for dimethachlor (data not shown) and metolachlor respectively. For atrazine, no value was available for ESLE because the culture failed. The exposure of each species to a wide range of concentrations of each herbicide did not lead to 100% inhibition of growth in all cases. The inhibition was usually between 80 and 100%.

**Table 3:** EC_50_ and EC_5_ (µg/l) values extrapolated from dose-response curves for 5 herbicides and 11 diatom species. 95% confidence intervals are shown in square brackets. A dash indicates a failed culture.

		Herbicides				
Species	ECx	Diuron	Atrazine	Isoproturon	Terbutryn	Metolachlor
*Craticula accomoda*	EC_50_	1734 [1578;1985]	919 [865;1011]	853. [808; 901]	814 [751; 877]	30147 [17134; 44657]
	EC_5_	261 [210; 292]	524 [514;534]	277 [251; 294]	92[72; 113]	2575 [1729; 2999]
						
*Eolimna minima*	EC_50_	4236 [3905; 4529]	2510 [2313;2748]	1566 [1536; 1594]	3133 [2728; 3512]	>50000
	EC_5_	3007 [2809; 3173]	1443 [1397;1499]	747 [611; 1139]	1450 [1211; 1790]	
						
*Mayamaea fossalis*	EC_50_	463 [311; 658]	8297 [7893;8900]	1664 [1625;1713]	63 [61; 67]	10313 [8510;12020]
	EC_5_	74 [62;89]	4766 [3861; 5389]	559 [546; 576]	13 [10]; [18]	3393 [1791; 4389]
						
*Encyonema silesiacum*	EC_50_	8.79 [7.51;9.88]	-	44 [42]; [47]	5.15 [4.26; 6.30]	6399 [5946; 6522]
	EC_5_	3.11 [2.10;4.21]	-	12.51 [11.26; 13.47]	0.55 [0.40;0.78]	54 [52; 56]
						
*Gomphonema parvulum*	EC_50_	2255 [1920; 2518]	907 [837; 995]	1014 [962; 1065]	464 [409; 564]	4054 [3397;4384]
	EC_5_	904 [739; 1144]	588 [554; 633]	652 [505; 892]	22 [20]; [26]	299 [160; 391]
						
*Fragilaria capucina* var *vaucheriae*	EC_50_	4.03 [3.93; 4.16]	801[689; 966]	117 [98; 131]	60 [59; 62]	>50000
	EC_5_	0.069 [0.062; 0.073]	205 [125;287]	35 [31]; [38]	31 [28]; [34]	
						
*Fragilaria ulna*	EC_50_	51 [48; 54]	306 [289; 318]	74.38 [65.91;85.24]	56 [45; 60]	3314 [2609; 3570]
	EC_5_	12.6 [11.37; 13.0]	191 [173; 207]	30 [27]; [33]	0.85 [0.73; 0.95]	60 [52; 68]
						
*Fragilaria rumpens*	EC_50_	122 [117; 127]	629 [596; 756]	357 [319; 450]	477 [457; 497]	>50000
	EC_5_	18 [13]; [20]	210 [164;304]	22 [18]; [30]	0.70 [0.51; 0.90]	
						
*Nitzschia palea*	EC_50_	1539 [1336; 1697]	3988 [3519; 4467]	1577 [1364; 1881]	1414 [1186; 1663]	>50000
	EC_5_	106 [77; 129]	596 [411;798]	222 [189; 271]	81 [41; 108]	
						
*Achnanthidium minutissimum*	EC_50_	108 [89; 137]	748 [562;888]	173 [150; 205]	411 [372; 447]	8551 [7067; 9853]
	EC_5_	3.15 [2.61; 3.87]	129 [109;143]	24.78 [16.83; 24.90]	63 [58; 69]	5957 [5573; 6236]
						
*Cyclotella meneghiniana*	EC_50_	23 [21]; [25]	812 [726;908]	46 [43]; [48]	9.62 [9.39; 9.97]	3476 [2582; 4042]
	EC_5_	1.59 [0.90; 2.05]	58 [45;67]	3.16 [2.57; 3.64]	5.38 [5.21; 5.48]	1282 [1084; 1443]

The 11 species of diatoms tested displayed very variable sensitivity for the same herbicide at the EC_5_ and EC_50_ levels (Table 3). Phenylurea EC_50_ values varied from 4.03 µg/l (FCVA) to 4236 µg/l (EOMI) for diuron and from 44 µg/l (ESLE) to 1664 µg/l (MAFO) for isoproturon. Triazines EC_50_ ranged from 306 µg/l (FULN) to 8297 µg/l (MAFO) for atrazine and from 5.15 µg/l (ESLE) to 3133 µg/l (EOMI) for terbutryn. Finally, metolachlor EC_50_ varied from 3314 (FULN) to over 50000 µg/l (NPAL, EOMI, FRUM). EC_5_ values derived from dose-response curves for FCVA exposed to diuron and FULN exposed to terbutryn were below the lower concentration tested.

### Species sensitivity patterns

**Figure 1:**
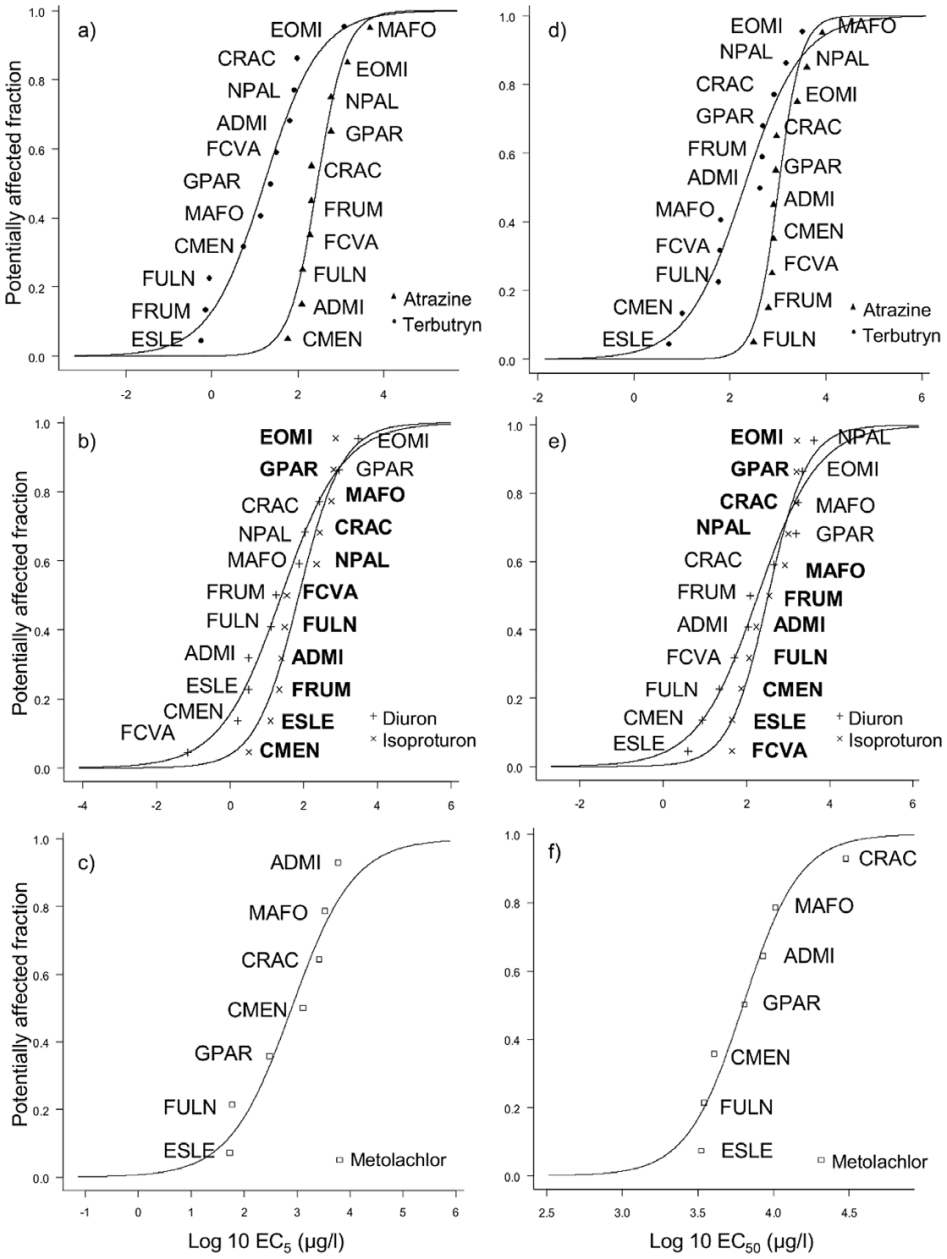
SSD curves of the 5 herbicides and details of species sensitivity ranking. Curves are based on EC_5_ values a), b), c), and EC_50_ values d), e), f) for five herbicides grouped according to their mode of action. In b) and e) the code name in bold type corresponds to the isoproturon curve.Species references: FCVA: *Fragilaria capucina* var. *vaucheriae*; FRUM: *Fragilaria rumpens*; FULN: *Fragilaria ulna*; CRAC: *Craticula accomoda*; MAFO: *Mayamaea fossalis*; EOMI: *Eolimna minima*; NPAL: *Nitzschia palea*; ADMI: *Achnanthidium minutissimum*; CMEN: *Cyclotella meneghiniana*; ELSE: *Encyonema silesiacum*; GPAR: *Gomphonema parvulum*.

EC_5_ and EC_50_ values obtained from dose-response curves were used to plot SSD-EC_5_ and SSD-EC_50_ curves (Fig. 1). HC_5_, HC_50_ and slope values were obtained from SSD-EC_5_ (SSD-EC_5 HC5_ or SSD-EC_5 HC50_) and SSD-EC_50_ (SSD-EC_50 HC5_ or SSD-EC_50 HC50_) (Table 4). From SSD-EC_5_ to SSD-EC_50_, the curves shifted toward higher concentrations, due to the higher values of EC_50_. On SSD-EC_5_ and SSD-EC_50_ curves of atrazine, terbutryn, diuron and isoproturon, the least sensitive species were always EOMI, GPAR, NPAL, CRAC and MAFO (except for terbutryn). For SSD-EC_50_ curves of all PSII inhibitors, FCVA, FULN, CMEN and ELSE were always the most sensitive species among species of the assemblage. For metolachlor, CRAC, MAFO, ADMI and GPAR were the least sensitive species, while CMEN, FULN and ELSE were more sensitive.

**Table 4:** Hazardous concentration (HC_5_ and HC_50_) values extrapolated from SSD curves based on EC_5_ or EC_50_ of diatoms for 5 herbicides.

	SSD-EC_5_		SSD-EC_50_	
Herbicide	HC_5_ (µg/l)	HC_50_ (µg/l)	HC_5_ (µg/l)	HC_50_ (µg/l)
Diuron	0.09	24.83	1.43	188.21
Isoproturon	1.38	73.46	13.90	332.24
Atrazine	29.90	283.52	202.91	1020.88
Terbutryn	0.21	16.48	3.33	195.2
Metolachlor	14.93	787.69	1784.36	6312.69

### Order of toxicity between herbicides

**Figure 2:**
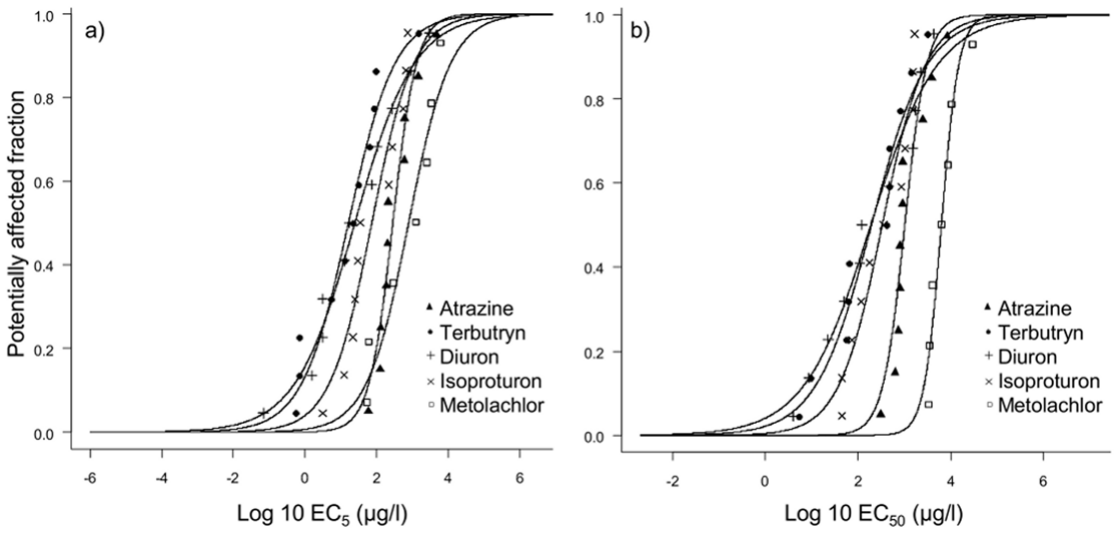
SSD curves of each herbicide based on benthic diatom sensitivities. Curves are based on a) EC_5_ and b) EC_50_ values. Each symbol represents one species.

According to the HC_5_ values extracted from SSD-EC_50_, the toxicity ranking of the herbicides was: diuron (1.43 µg/l) > terbutryn (3.33 µg/l) > isoproturon (14 µg/l) > atrazine (203 µg/l) > metolachlor (1784 µg/l) (Table 4, Fig. 2). We found the same ranking for other HC_x_ values of SSD-ECx, with the exception of SSD-EC_5 HC50_, where terbutryn was slightly more toxic than diuron.

## Discussion

### Ecotoxic responses of benthic strains

In this study, diatoms of benthic origin were cultured as plankton, which mean that the biofilm structure and its possible consequences for the responses of the microalgae were not taken into account. It has in fact been shown that the biofilm can have a protective effect, or increase the exposure of the diatoms [37]. Consequently, the sensitivity level of the diatoms calculated here usually reflects the specific physiological characteristics of the strains tested rather than the effect of the biofilm on exposure to the toxic substance. Depending on its mechanism of action, each herbicide produces a specific toxic effect on these species, and the toxicity rankings found in this study are similar to those already observed for microalgae and/or macrophytes [27], [36], [38]. PSII inhibitors seem to inhibit diatom growth more effectively than herbicides with other modes of action, but a considerable difference in toxicity was found between atrazine and diuron. Their mechanism of action involves competing with plastoquinone so as to block the flux of electrons in the thylakoid membrane, thus leading to the dissipation of light energy [10]. As photosynthesis is the key functional process in primary producers, inhibiting this process more readily affects the growth of algae than inhibiting other functions. Certain herbicides in the chloroacetamide family, such as metolachlor, block the germination of seeds by inhibiting the synthesis of the very long chain fatty acids. In microalgae, this inhibition can impair the stiffness and permeability of the plasma membrane, resulting in inhibition of cell division [11], [15], [39]. Some studies have also demonstrated the teratological potential of metolachlor for diatoms, and its ability to increase the volume of the cells [14], [15]. The herbicides in the sulfonylurea family are used in agriculture to inhibit the growth of higher plants. They act by eliminating the biosynthesis of amino acids via inhibition of the enzyme acetolactate synthase, which implies that they have true phytotoxic potential [12]. Some authors have worked on the toxicity of various different herbicides belonging to this group (e.g.: metsulfuron methyl, nicosulfuron, chlorsulfuron) and have demonstrated considerable variation in the toxicities of these substances towards phytoplanktonic species [12], [40].

Overall, the EC_50_ values obtained in this study were very high compared to the concentrations of herbicides found in the environment, and it is therefore unlikely that the *in-situ* exposure levels would produce drastic effects on the diatom species tested. However, the EC_5_ values are sometimes fairly close to the concentrations encountered in the environment (e.g.: terbutryn [<1 µg/l], diuron [<3 µg/l]) for some strains, such as FCVA, CMEN, ESLE, FRUM, and FULN. These species are associated with high values of the sensitivity index in the context of the generic index for diatoms [8]. In contrast, Rimet and Bouchez [41] have shown ESLE to be relatively resistant during its benthic phase, and they suggest that the fact that this species has a mucous tube might protect the cells when they develop in a biofilm. However, no structure corresponding to a mucous tube was observed in this strain in our study.

The EC_50_ and EC_5_ values calculated from the dose-response curves have demonstrated considerable variability in the sensitivity of the strain, regardless of the herbicide being tested. However, the general pattern of the sensitivity of the species, revealed by the SSD-EC_50_ curves, does seem to be similar for all four PSII-inhibitor herbicides. The biological and ecological characteristics of the diatoms are beginning to be well attested [8], [9], [42], but little work has been done to relate the ecological characteristics of the diatoms to their sensitivity towards toxic agents [14], [40]. For the PSII inhibitors, the 4 least sensitive species all share the same trophic mode: they are all heterotrophs, either obligatory (CRAC, NPAL) or optional (mixotrophs, such as EOMI, GPAR) depending on their Van Dam trophic index [9]. These strains are likely to be less affected by PSII inhibitors than autotrophic species, because they are less dependent on photosynthesis products for their growth. The other more sensitive species (with an EC_50_ below the median) are all autotrophs, with the exception of CMEN. Facultative heterotroph diatoms can change their trophic mode according to changes in environmental conditions, which permits better adaptation [43]. Some authors have noted the presence or even dominance of heterotrophic species in the context of PSII inhibitor exposure as a result of adaptation and selection processes. Debenest et al. [29] showed an increase in the abundance of facultative heterotrophic diatoms after exposure to 30 µg/l of isoproturon. Pérès et al.[44], observed that some of the tolerant species were heterotrophic, such as *GPAR* and *Sellaphora seminulum*. An increase in the relative abundance of heterotrophic and tolerant autotrophic species was observed by Ricart et al. [45] following diuron exposure.

It looks as though the tolerance to PSII inhibitors of these species may also be correlated with the 3 ecological guilds defined by Passy [42], the taxonomic list of which has been enhanced by Berthon et al. [46]. The “high profile” guild (in our tests GPAR, and *Fragilaria spp*.) includes large-size species that are advantaged by weak flow velocity, in contrast to the “low profile” guild (in our tests: *ADMI, CMEN*, and *ESLE*), which consists of small-sized species able to withstand flow velocity as a result of their position in the lower part of the biofilm and encompass slow-moving species. The “motile” guild (in our tests *CRAC, NPAL, EOMI* and *MAFO*) which includes species capable of moving around within the biofilm display the greatest tolerance in our tests, regardless of the pesticide tested. All of *Fragilaria* species (FCVA, FULN, FRUM) in this study show high sensitivity to PSII inhibitors that seem to be correlated to theirs common guild. Fragilariales order is one of the most diverse for benthic diatoms in terms of life forms [47]. It encompasses many species with different morphological and physiological characteristics. For this reason, Berthon et al. [46] have shared this order between high and low profile guild. In our study, the three different species of *Fragilaria* we worked on all belong to the “high profile” guild. Two of them, FCVA and FRUM, have the same shape, length, biovolume, and ecological preferences, while FULN have cells which are about ten times longer and present a biovolume more than 100 times larger [48]. It is important to highlight that GPAR, which is one of the most resistant species of this study belong to this “high profile” guild too. Contrary to *Fragilaria* species, GPAR have heterotroph capabilities which may confer it a higher resistance to herbicide contamination.

Rimet and Bouchez [41] observed a reduction in the abundance of the species of the “high profile” guild, which is the opposite of the situation for those in the “motile” guild, and they attribute this to the fact that the “high profile” species may face greater exposure to dissolved substances. Our findings therefore establish a link between the trophic mode and the response of the strains to the PSII inhibitors. However, this hypothesis is not applicable to the herbicides in this study that display some other mode of action. In the case of metolachlor, Roubeix et al. [14] have observed a reduction in the abundance of ADMI, NPAL and *Mayamaea permitis* at 30 µg/l, whereas the values of the LOEC and EC_50_ for ADMI obtained with their single-species bioassay are much higher (180 and 1880 µg/l respectively). Debenest et al. [29] also demonstrated a reduction in the growth rate via chlorophyll c and the cell density at 30 µg/l of metolachlor in the periphyton. Other more pertinent and sensitive endpoints than the growth or the biomass, such as the level of very long-chain fatty acids, would provide complementary information for the assessment of the toxicity of the chloroacetamides. Finally, too little information is available about the toxicity of amidosulfuron and of foramsulfuron to make it possible to determine the effects of these herbicides on diatoms. What is certain, is that the cellular mechanisms targeted by the sulfonylureas and the chloroacetamides have less effect on the growth of benthic diatoms than the photosynthesis targeted by PSII inhibitors.

### Risk evaluation and SSD models

Analysis of the EC_50_ has revealed that there is considerable variability for a given substance depending on the strain considered. The basic assumptions underlying SSD is that different species can display differing sensitivities towards the same toxicant in the environment. The SSD model allowed us to demonstrate this variation of sensitivity among an assemblage using data obtained by means of single-species bioassays [20] and, *in fine*, to extrapolate the HC for a natural community, which is more realistic from an environmental standpoint. Our SSD curves were based on sensitivity data for 11 diatom species obtained using the same protocol. Wheeler et al [34] recommended using more than 10 data in order to obtain a more reliable HC. The HC_5_ values obtained in our study and determined from the SSD-EC_50_ and SSD-EC_5_ curves are very variable, and tend to be higher in the case of metolachlor and atrazine. However, the fact that this modeling was however carried out for single-species, implies that the distribution ignores any interaction between the species [20], [49]. In this study, the equations used to establish the SSDs do indeed model the differences in sensitivity of the diatoms within the reconstituted assembly, and the level of toxicity of each herbicide. To ensure environmental pertinence, Forbes and Calow [50] recommend carrying out SSDs from species that are representative of the environment being studied. The diatoms selected for this study are not all strictly associated with a benthic life form, but they are representative and cover the coastal zones as well as providing wide diversity in terms of taxonomy, of sensitivity and ecological traits. The HC_5_ values that we determined from our SSD-EC_50_ and SSD-EC_5_ curves vary considerably for different herbicides, and tend to be higher in the case of metolachlor and atrazine.

Some studies have used scientific literature and toxicity databases to construct the SSD curves [1], [27], [36], but the amount of data available varies considerably depending on the substances. In general, the paucity of toxicity data hinders the construction of specific curves for a functional group and for a given substance, resulting in curves that are not very robust, based on too few data or on pooled groups and differing endpoints. Some authors have constructed SSD-EC_50_ curves for atrazine, isoproturon or diuron based on the sensitivity of primary producers including macrophytes and microalgae [1], [36]. The various HC values extrapolated from these curves were much lower than those we found in our study. The SSD-EC_50_ curves based on the sensitivity of several primary producers, not only benthic organisms, plotted by Schmitt-Jansen and Altenburger [27] revealed greater sensitivity of the assemblages towards atrazine and isoproturon than in our study. This means, that the HC values obtained from SSD curves based on various endpoints and using phototrophic organisms are lower than those specifically involving benthic diatoms. These differences could be explained by the differences in the sensitivities of the algal classes towards the PSII inhibitors or to compounds with other mechanisms of action and by the species composition. Guasch et al [43] observed that periphyton communities in which diatoms dominated were more resistant to atrazine than those dominated by green algae. Specific SSD curves based on chlorophyte and diatom sensitivities to atrazine are different in shape [27], and the median EC_50_ found for diatoms was more than twice as high as those found for chlorophytes. The fact that benthic diatoms seemed more resistant to PSII inhibitors than chlorophytes led to a threshold not enough protective for chlorophytes wich make up small proportions of biofilms.

For diuron and terbutryn, these HC_5_ values correspond to concentrations sometimes found in water column. In France, peaks of about 3 µg/l of diuron were found in 2009 in superficial water of some stations of Adour-Garonne catchment, [7].In other European river, peak level of isoproturon and diuron of 1,96 and 0.86 µg/l, respectively, have been reported [2]. HC5 obtained from SSD-EC5 for these two herbicides are lower than these environmental concentrations found in water. Finally, the AA-EQS of diuron defined as 0.2 µg/l, exceed the SSD-EC5 HC5 value. In view of our results, this implies that the concentrations of diuron and isoproturon found in the environment carrya potential risk of reducing diversity in communities of benthic diatoms. However terbutryn is not a prioritary substance, and we can say that in the light of its low HC5, very low concentrations of this substance can affect benthic diatom communities. Finally, as SSDs based on EC_5_ values gave HC5 values below the environmental concentration, curves based on these thresholds are probably more appropriate to ensure that benthic diatom communities are protected.

Single-species bioassays of these 11 species revealed the huge variation of sensitivities present in a given algal group for the same herbicide. The main finding to emerge is mainly that the trophic mode plays an important role in the resistance of benthic diatoms towards PSII inhibitors. For risk assessment, SSD curves based on log-logistic regression fitted well. If the panel of algal strains and the endpoint selected to some extent determine the final HC_5_, this study has demonstrated that diuron, isoproturon and terbutryn, even at low levels, constitute a risk for natural communities of benthic diatoms. In view of these results, risk assessments of herbicides for the total biofilm algal community should be done using specific SSD curves for each of the algal groups present in biofilm or in the phytoplankton communities.

In addition to performing studies, improving risk assessment involves taking the real *in-situ* exposure levels facing these benthic diatoms into account, i.e. taking into account the mixtures of substances. The trials are currently under way to include these factors in the evaluation of the risks linked to the pesticides in aquatic ecosystems.
